# Accumulation and transmission dynamics of ‘*Candidatus* liberibacter solanacearum’ haplotypes A and B by potato psyllid nymphs: bioassay and transcriptomic insights

**DOI:** 10.1007/s11033-025-11417-y

**Published:** 2026-01-06

**Authors:** Junepyo Oh, Azucena Mendoza Herrera, Brenda Leal-Galvan, Aidan Burnett, Cecilia Tamborindeguy

**Affiliations:** 1 Department of Entomology, Texas A&M University, College Station, TX 77843 USA; 2 Department of Horticulture Sciences, Texas A&M University, College Station, TX 77843 USA

**Keywords:** Bactericera cockerelli, Liberibacter, Insect-microbe interaction, Immunity, Tomato, RNA-seq

## Abstract

**Background:**

‘*Candidatus* Liberibacter solanacearum’ (Lso) is a phloem-limited bacterial pathogen causing significant diseases in solanaceous crops. In the United States, haplotypes A and B are transmitted by the potato psyllid *Bactericera cockerelli*. We previously identified differences in their acquisition and transmission between adults and nymphs. The present study characterized the dynamics of LsoA and LsoB acquisition and transmission by nymphs and examined the transcriptional responses of the nymphal gut upon their acquisition.

**Methods and results:**

Nymphs were exposed to LsoA- or LsoB-infected plants for 1, 3, 5, or 7 days to measure the bacterial accumulation and for 8 days to assess the transmission efficiency following sequential inoculation of tomato plants. Quantitative PCR showed that LsoB accumulated to higher levels than LsoA after 3 days of acquisition. Following the sequential inoculation, LsoB was transmitted earlier than LsoA indicating a shorter latency period. RNA-seq analysis of the guts following a 1- and 5-day acquisition access periods revealed a greater transcriptional regulation at 5 days than at 1 day. Furthermore, the responses were haplotype-specific: LsoA primarily affected genes involved in protein translation, ER stress, and cell cycle regulation, whereas LsoB regulated genes involved in autophagy, apoptosis, and immune pathways.

**Conclusions:**

This study revealed haplotype-specific gene regulation potentially leading to LsoB being transmitted more efficiently by psyllid nymphs.

**Supplementary Information:**

The online version contains supplementary material available at 10.1007/s11033-025-11417-y.

## Introduction

‘*Candidatus* Liberibacter solanacearum’ (Lso) is a Gram-negative, phloem-limited bacterial pathogen that causes significant damage to crops globally [1]. More than ten haplotypes of Lso have been reported [2–11]. In the United States, LsoA and LsoB are transmitted by the potato psyllid *Bactericera cockerelli*, also known as the tomato psyllid. Both haplotypes infect solanaceous crops such as tomatoes, and cause diseases including zebra chip in potatoes, leading to major yield and quality losses [12–14]. Several studies have reported differences in pathogenicity between LsoA and LsoB: LsoB kills tomato and tobacco plants while LsoA does not [13, 15], and LsoB causes higher nymphal mortality [16]. Importantly, differences in the acquisition and transmission of these two haplotypes by potato psyllid adults were also identified. LsoB titer increased more rapidly than LsoA in the gut of potato psyllid adults, and it was transmitted more efficiently [17]. These findings suggest that the two haplotypes induce different responses in host plants and insect vectors.

Potato psyllid nymphs can transmit Lso as early as third-instar with a short inoculation access period (IAP), when harboring both LsoA and LsoB [18]. More recently, we showed that third-instar nymphs from Lso-infected colonies accumulated higher LsoA than LsoB titers in their gut and could transmit LsoA but not LsoB, whereas fifth-instar nymphs had similar titers of both haplotypes and transmitted both [19]. These findings were surprising as they were opposite to those obtained when studying the accumulation of Lso upon acquisition by adults [17]. Because adults are highly mobile and nymphs remain on the same host and potentially have a weaker immune system, differences in pathogen-vector interactions between life stages are expected [20].

We had also previously shown that LsoB infection resulted in increased nymphal mortality [16] and that the higher the Lso titer, the greater the mortality [21]. Therefore, differences in the accumulation and transmission of these haplotypes by nymphs could result from acquisition dynamics, mortality associated with high LsoB titers, or trans-generational immune priming in nymphs whose mothers are infected [22]. To better understand these differences, we evaluated the accumulation and transmission of LsoA and LsoB by nymphs upon acquisition, complementing our previous study with nymphs continuously reared on infected plants [19]. We also compared the gut transcriptome of nymphs after feeding on LsoA- or LsoB-infected tomato plants for 1 and 5 days, corresponding to early and later stages of gut infection. Our main goals were to evaluate the acquisition and transmission of LsoA and LsoB by nymphs and to identify gut transcriptional responses to each haplotype. This work complements our earlier transcriptomic analysis of adult psyllid guts and advances our understanding of psyllid-Lso interactions and stage-specific responses, with implication for the epidemiology of Lso disease.

## Materials and methods

### Insect colonies and plants

Lso-free, LsoA-, and LsoB-infected potato psyllid colonies were kept in insect-proof cages (24 by 13.5 by 13.5 cm; BioQuip, Compton, CA) on tomato plants at 22 ± 2 °C under 16:8 h light: dark. Diagnostic end-point PCR tests were conducted regularly to detect Lso and determine the haplotype using LsoF/012 for Lso detection and SSR1 for haplotype determination [23, 24].

*Solanum lycopersicum* L. ‘Moneymaker’ seeds (Victory Seed Company, Irving, TX) were planted in Sun Gro Sunshine LP5 mix (Bellevue, WA), and maintained under the same conditions. To obtain Lso-free, LsoA- and LsoB-infected plants, five male adults from each colony were caged for one week in a mesh bag on 4-week-old tomato plants. Four weeks after insect removal, young top leaves were collected to confirm Lso infection and haplotype by diagnostic PCR. DNA extraction from leaf tissue was performed using the CTAB method following the protocol previously described by J. Levy et al. [25].

### Lso accumulation in nymphs

Third-instar nymphs from a Lso-free colony were allowed a 1-, 3-, 5-, and 7-day acquisition access period (AAP) on LsoA- and LsoB-infected plants. After each AAP, ten nymphs from the same group were pooled and DNA was extracted as in Nachappa et al. [26]. Lso titer was quantified by absolute qPCR using a QuantStudio 6 Flex Real-Time PCR System (Applied Biosystems, Waltham, MA). Each 10 µL total reaction contained 25 ng of DNA, 250 nM final concentration of each primer, and 1X SYBR master mix (Applied Biosystems, Foster City, CA). The 16 S rDNA Lso-specific primers (LsoF: 5′-CGAGCGCTTATTTTTAATAGGAGC-3′ and HLBR: 5′- GCGTTATCCCGTAGAAAAAGGTAG-3′) [23, 27] and psyllid 28 S rDNA primers (28 S rDNAF: 5′-AGTTTCGTGTCGGGTGGAC-3′ and 28 S rDNAR: 5′-AACATCACGCCCGAAGAC-3′) [21] were used. The qPCR program was 95 °C for 2 min and 40 cycles at 95 °C for 5 s followed by 60 °C for 30 s. Each qPCR had two technical replicates and a negative control. To standardize Lso titer, a standard curve was generated using the Lso 16 S rDNA cloned into TOPO TA vector as described by J. Levy et al. [25]. The resulting curve had a slope of -3.45, y-intercept of 34.84, and R^2^ = 0.998, corresponding to a qPCR efficiency of 94.9%. All raw Ct values for the standard and samples are provided in Supplementary Table S1. The absolute Ct values of 16 S rDNA were converted to copy numbers using the standard curve. Three pools (biological replicates) of 10 nymphs for each AAP and Lso haplotype combination were analyzed; each pool was collected from a different plant. The experiment was independently repeated three times.

### Lso transmission by nymphs

To evaluate the transmission efficiency of LsoA and LsoB by psyllid nymphs, Lso-free third-instar nymphs were exposed to a LsoA- or LsoB-infected tomato plant for an 8-day AAP. Afterward, 10 fifth-instar nymphs per treatment were individually transferred to 10 4-week-old non-infected tomato plants for a 9-day inoculation access period (IAP), consistent with the Lso latent period [28]. The remaining nymphs were maintained on non-infected tomato plants and used as replacements in case nymphs died during the assay. Subsequently, every four days, each nymph was transferred to a new non-infected recipient plant. The sequential transmission was discontinued on day 29 (three transfers) due to increasing psyllid mortality. Plant infection was tested 6 weeks after psyllid removal and, if negative, again at 8 weeks. The transmission assays were performed twice independently.

### Gut transcriptome sequencing following Lso acquisition

Third-instar nymphs from the Lso-free colony were allowed a 1- and 5-day AAP on Lso-free, LsoA-, and LsoB-infected tomato plants. After each AAP, guts were dissected under a Leica EZ4W0037 stereomicroscope (Leica Microsystems, Wetzlar, Germany) using a 1:1 mixture of RNAlater (Thermo Fisher Scientific, Waltham, MA) and 1X phosphate-buffered saline (PBS). Fifty dissected guts from the same group were pooled in RNAlater/1X PBS and placed at 4 ˚C for 24 h. Each pool represented one replicate; different plants were used for each pool. There were three biological replicates for each AAP (1- and 5-day) and Lso treatment (Lso-free, LsoA, and LsoB), for a total of 18 samples. RNA was extracted using the RNeasy Mini Kit (Qiagen, Valencia, CA) and treated with TURBO DNA-free Kit (Thermo Fisher Scientific). RNA quality assessment, cDNA library construction, and sequencing were performed at the Texas A&M AgriLife Genomics and Bioinformatics facility. The libraries were obtained using the SMARTer ultra-low RNA kit for Illumina sequencing (Takara Bio, San Jose, CA) and sequenced on NovaSeq 6000 S2 platform (150 bp paired-end reads). Raw reads underwent quality control and preprocessing by the sequencing facility as part of their standard Illumina pipeline. FastQC results indicated that the sequence quality met recommended thresholds, and the processed reads were then used for downstream analyses.

### Bioinformatic analyses

The transcriptome was analyzed in the CyVerse Discovery Environment using the Kallisto-Sleuth pipeline. We generated a transcript index using Kallisto with 50 bootstraps and the potato psyllid transcriptome and calculated transcript abundances [29]. Differential expression analysis was performed with Sleuth [30], and differentially expressed genes (DEGs) were identified using the Wald test (q-value < 0.05). Gene expression was compared among the different infection treatments at each AAP (1- and 5-day AAP). Venn diagrams were generated using the Venny 2.1 online web tool (https://bioinfogp.cnb.csic.es/tools/venny/*)* to identify unique and shared DEGs. To better understand the biological relevance of the gene regulation, the DEGs were annotated using Blast2GO version 6.0.3 and Fisher’s exact test (FDR < 0.05) was used to conduct GO term enrichment analyses. Bar plots of the proportion of sequences associated with enriched GO terms were created using R (v4.3.1) and ggplot2. GO terms were grouped into biological process (BP), cellular component (CC), and molecular function (MF) and panels combined using the patchwork package in R (v4.3.1) [31].

### Validation of transcriptome by real-time quantitative PCR (RT-qPCR)

For validation, third-instar nymphs from the Lso-free colony were allowed a 5-day AAP on Lso-free, LsoA-, and LsoB-infected tomato plants as described above. There were three plants per infection type. After the AAP, guts were dissected and pooled by treatment; each pool had 30 guts. These samples were obtained independently from those used for RNA-seq. RNA purification and DNase treatment were performed as previously described. Two hundred nanograms of RNA from each sample were used to synthesize cDNA using the Verso cDNA Synthesis kit (Thermo Fisher Scientific). Each qPCR reaction (10 µL total volume) contained 10 ng of cDNA, 250 nM final concentration of each primer (Table S2) and 1X SYBR master mix (Applied Biosystems). The qPCR program was described earlier in the methods section. Three technical replicates and negative controls (lacking cDNA) were included in each run. The relative expression was evaluated using 2^^^-ΔΔCT method (Schmittgen & Livak, 2008), with Lso-free as the control group and ribosomal protein subunit 18 (GenBank KT279693) as a reference gene [32], and subjected to a log_2_-transformation.

### Statistical analysis

qPCR data were analyzed using JMP Version 16 (SAS Institute Inc., Cary, NC) and GraphPad Prism 9.5.1 Software (GraphPad Software, San Diego, CA, USA). The LsoA and LsoB titers were log_10_ transformed for the analysis to ensure normality. Homogeneity of variance was verified using Levene’s Test and the normality of residuals using the Shapiro-Wilk test. Because the experiment included four discrete, fixed AAP time points, AAP was treated as a categorical factor. A two-way analysis of variance (ANOVA) was used to test the effects of Lso haplotype and AAP, followed by Tukey’s post hoc test.

Transmission data were analyzed using R (https://www.r-project.org/). For each time point, a 2 × 2 contingency table was constructed with the numbers of infected and non-infected plants in the LsoA and LsoB groups. Differences in infection frequency were evaluated using Fisher’s exact test as implemented in the fisher.test() function of R v4.3.3. This test computes an exact two-sided P-value based on the hypergeometric distribution and is recommended for small sample sizes. The odds ratio (OR) was calculated to quantify the relative likelihood of infection between the two groups. The OR was determined using the formula:

$$\:Odds\:ratio\:\left(OR\right)=\:\frac{a\:\times\:\:d}{b\:\times\:\:c}$$

Where *a* and *b* represent the number of infected and non-infected plants in the LsoA group, respectively, and *c* and *d* indicate the infected and non-infected plants in the LsoB group.

The 95% confidence interval (CI) for each OR was obtained using the profile-likelihood method provided by fisher.test(). When a cell count was zero, Haldane–Anscombe continuity correction (adding 0.5 to all cells) was applied to permit OR estimation. Statistical significance was accepted at *p* < 0.05, and both *p*-values and OR ± 95% CI are reported for each day.

## Results

### LsoB accumulates faster than LsoA in nymphs

**Fig. 1:**
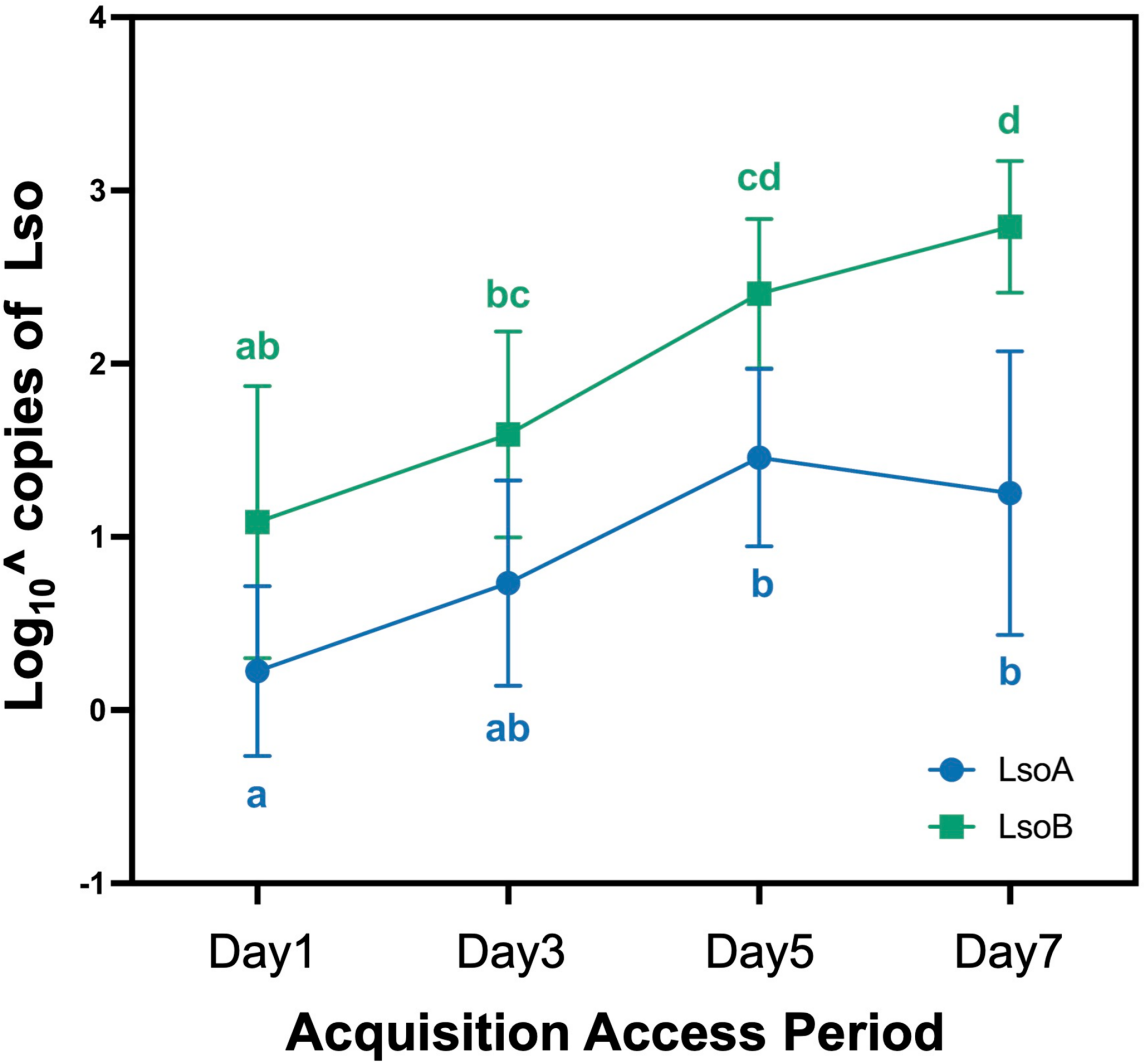
Quantification of Lso copies in potato psyllid nymphs following Lso acquisition. LsoA (dark blue) and LsoB (dark cyan) titers in pools of 10 nymphs following a 1-, 3-, 5-, and 7-day acquisition access period (AAP). Data represent means ± SD from three independent experiments, each with three biological replicates. Different letters indicate statistical differences at *p* < 0.05 using two-way ANOVA with Tukey’s post hoc test

Lso was quantified in pools of 10 nymphs following 1-, 3-, 5-, and 7-day AAPs on LsoA- or LsoB-infected plants. A two-way ANOVA indicated a significant effect of the haplotype [F(1, 64) = 56.0733, *p* < 0.0001] and the AAP [F(3, 64) = 21.5427, *p* < 0.0001] on the number of Lso genomes in the psyllid bodies. However, the interaction between these terms was not statistically significant [F(3, 64) = 1.3581, *p* = 0.2636]. Both LsoA and LsoB were detected after the 1-day AAP and LsoB titers were significantly higher than LsoA at the 5- and 7-day AAP. Although LsoA titer increased between the 1- and 5-day AAPs, it remained low during the observed AAPs (Fig. 1).

### LsoB completes its circulation faster than LsoA in nymphs

**Fig. 2:**
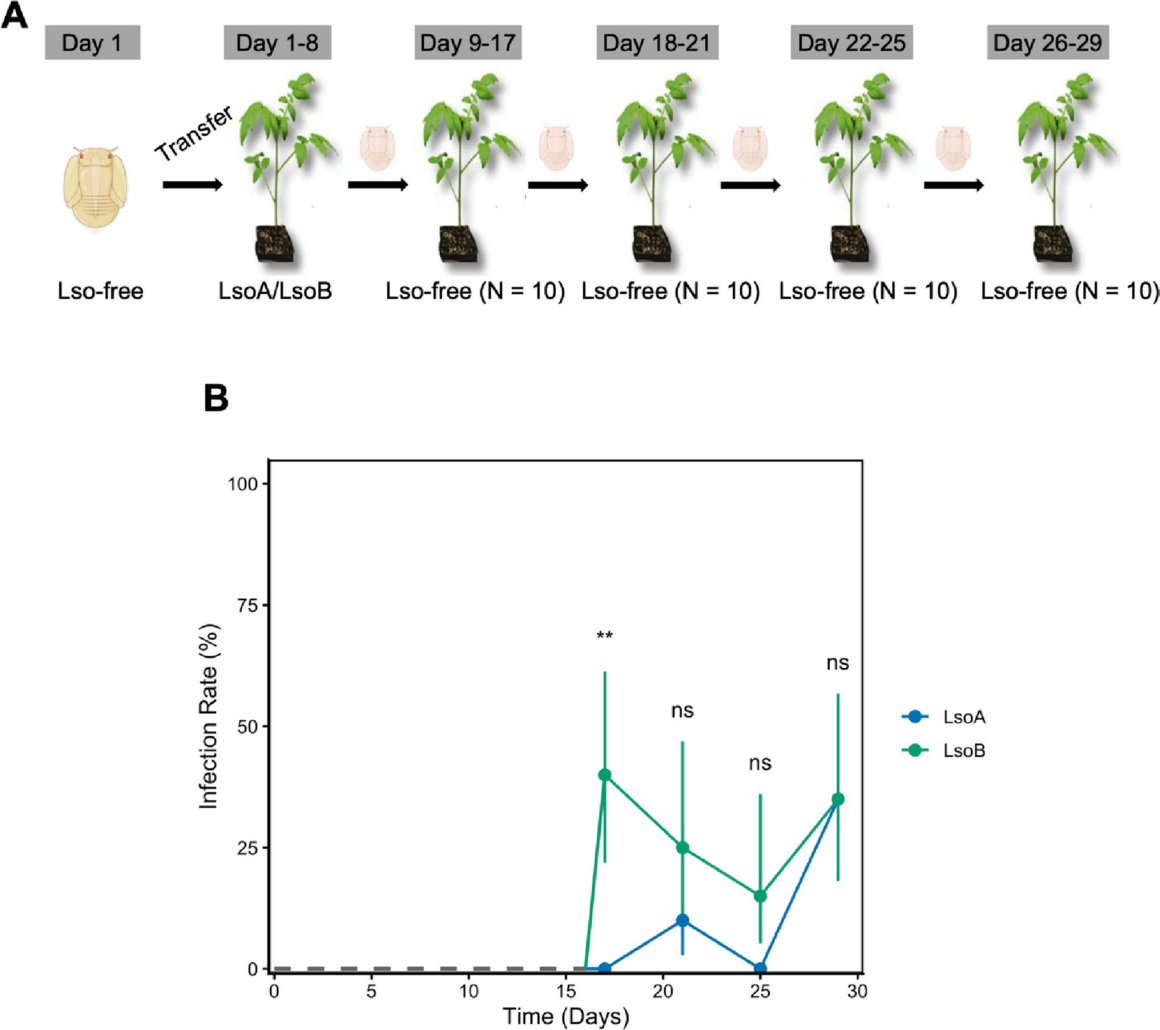
Transmission of LsoA or LsoB by psyllid nymphs. (**A**) Third-instar psyllid nymphs were given an 8-day AAP on Lso-infected tomato plants. Then, 10 psyllid nymphs per treatment were transferred individually to 10 recipient non-infected tomato plants for a 9-day inoculation access period to account for the transmission latency; after which, each psyllid was sequentially transferred to a new non-infected tomato plant every 4 days. The days shown in the figure represent the number of days following the beginning of the experiment: Day 1 corresponds to the day at which Lso-free psyllid nymphs were first placed on Lso-infected plants. (**B**) Transmission efficiency by psyllid nymphs from two independent experiments. The lines represent the mean infection rate (%) ± 95% Wilson confidence intervals (CI) for each group. A dashed gray line indicates the baseline (0% infection) prior to the first measurement. “**” indicate *p* < 0.01; “ns” indicates not significant

Because LsoB titer increased faster than LsoA in nymphs, we hypothesized that LsoB could also be transmitted earlier. To test this, we performed two independent sequential transmission experiments using tomato plants following an 8-day AAP (Fig. 2A). The results showed differences in the latent period and overall transmission efficiency between haplotypes (Fig. 2B and Table S3 and S4). In both experiments, LsoB was first detected in 40% of the recipient plants at day 17 (corresponding to an 8-day AAP and 9-day IAP to account for the transmission latency). No further transmission of LsoB was observed in the first experiment, which had high psyllid mortality: only three psyllids reached the end of the experiment alive without being replaced (Table S3). While we replaced the psyllids found dead when the host was switched, it is probable that the high mortality affected the overall transmission. In the second experiment, an average of 4.75 plants were infected by LsoB at each passage (Table S4). In contrast, LsoA infection was first detected at day 21 in experiment 1 (20% of plant infected), and at day 29 in experiment 2, but with higher efficiency (60%) (Table S4). Fisher’s exact test on combined experiments showed a significant difference in infection prevalence between LsoA and LsoB on Day 17 (*p* = 0.003, Table S5) with an odds ratio of 0.036 (95% CI: 0.002–0.677). This indicates that nymphs that acquired LsoA were significantly less likely to transmit the pathogen compared to those exposed to LsoB after a 17-day latency. No significant differences in infection rates were observed between haplotypes on days 21, 25, and 29.

### LsoA and LsoB have different effects on the nymph gut transcriptome

**Fig. 3:**
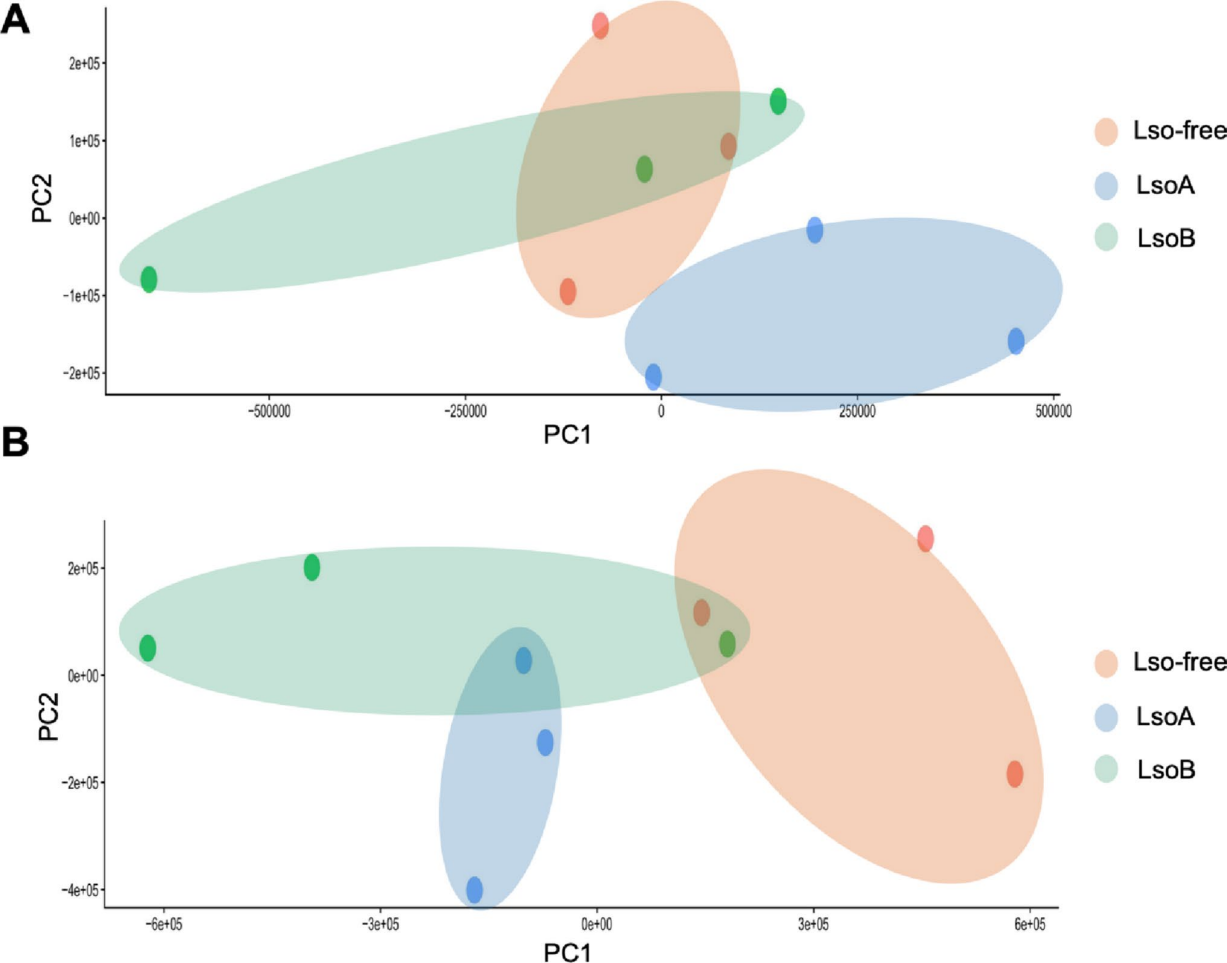
Principal component analysis (PCA) of the transcriptome samples (**A**) Nine libraries after a 1-day AAP on Lso-free, LsoA-, and LsoB-infected plants. (**B**) Nine libraries after a 5-day AAP on Lso-free, LsoA-, and LsoB-infected plants

We generated transcriptome datasets from pools of 50 guts from psyllid nymphs that were exposed to Lso-free (control), LsoA-, and LsoB-infected tomato plants for a 1- and 5-day AAP, respectively; six treatments in total. Three biological replicates were analyzed, resulting in 18 independent libraries with 20–27 million reads each (Table S6). A principal component analysis (PCA) did not show a good separation among the different treatments for the 1-day AAP samples, suggesting the few transcriptional changes occurred after short exposure (Fig. 3A). However, the libraries from the Lso-free and LsoA libraries clustered by treatment and could be separated, whereas LsoB libraries showed greater variability and overlapped with Lso-free libraries. A better separation among the treatments was observed for the 5-day AAP samples. Greater variation was still observed among the LsoB libraries. In particular, the PCA plot showed a relatively good separation between the infected and non-infected samples in the first dimension (except for one LsoB library, which may reflect lower Lso acquisition), with LsoA libraries clustering closer to Lso-free than to LsoB. Further, LsoA and LsoB samples were separated in the second dimension (Fig. 3B).

### Few genes were commonly regulated in the gut of nymphs by both Lso haplotypes

**Fig. 4:**
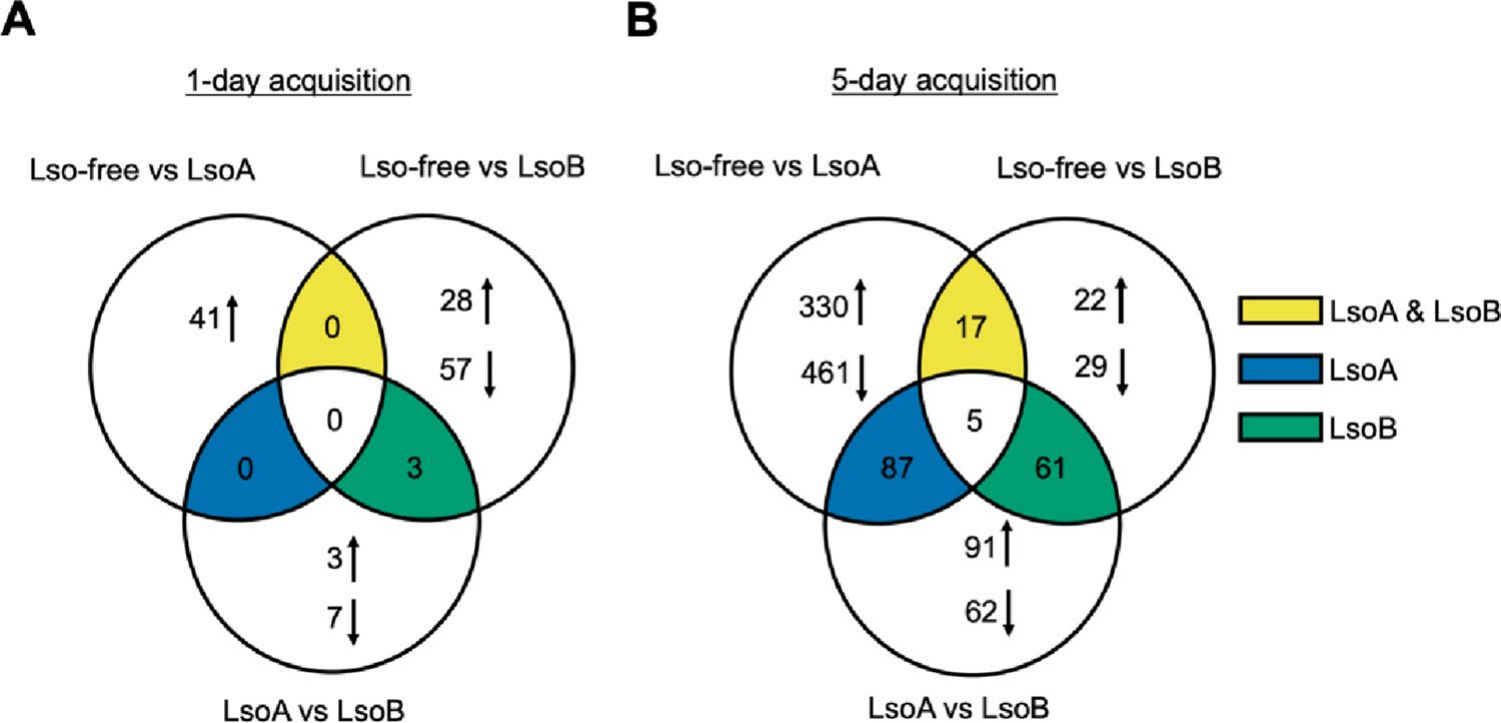
Venn diagram illustrating distinct and shared differentially expressed genes (DEGs). (**A**) DEGs identified after a 1-day AAP. (**B**) DEGs identified after a 5-day AAP. The bright yellow region indicates the DEGs shared between the Lso-free vs. LsoA and Lso-free vs. LsoB comparison. The dark blue region indicates the DEGs shared between the Lso-free vs. LsoA and LsoA vs. LsoB comparison. The dark cyan region indicates the DEGs shared between the Lso-free vs. LsoB and LsoA vs. LsoB comparison. The up and down arrows indicate the up- and down-regulation of DEGs in the Lso-infected treatment. For example, the up arrow between Lso-free vs. LsoA indicates gene up-regulation in the psyllid on LsoA-infected plants; in the LsoA vs. LsoB comparison, the arrows represent gene expression in LsoB compared to LsoA samples

We compared the transcriptomes to identify genes regulated in response to both Lso haplotypes; these genes represent core response genes. We also identified genes regulated in response to one of the haplotypes. After a 1-day AAP, 139 differentially expressed genes (DEGs) were identified (Fig. 4): 41 DEGs in the Lso-free vs. LsoA comparison (Table S7) and 88 DEGs in the Lso-free vs. LsoB comparison (Table S8), of which 30 were up-regulated and 58 (roughly two-thirds) were down-regulated in psyllids having fed on LsoB-infected plants. No DEGs were shared among the two comparisons. There were 13 DEGs identified in the LsoA vs. LsoB comparison (Table S9), three were common between the comparisons Lso-free vs. LsoB and LsoA vs. LsoB.

After a 5-day AAP, more DEGs were identified. There were 900 DEGs in the comparison of Lso-free vs. LsoA (Table S10): 361 were up-regulated and 539 were down-regulated in psyllids having fed on LsoA-infected plants. There were 134 DEGs in the Lso-free vs. LsoB comparison (Table S11): 84 up-regulated and 50 down-regulated DEGs in psyllids having fed on LsoB-infected plants. Only 22 DEGs were shared between the Lso-free vs. LsoA and Lso-free vs. LsoB comparisons (Table S12). In the LsoA vs. LsoB comparison, 306 DEGs were identified: 212 were up-regulated and 94 down-regulated in the LsoB treatment compared to the LsoA treatment.

### LsoA regulated translation and ER-related genes while LsoB regulated immune and stress response genes

**Fig. 5:**
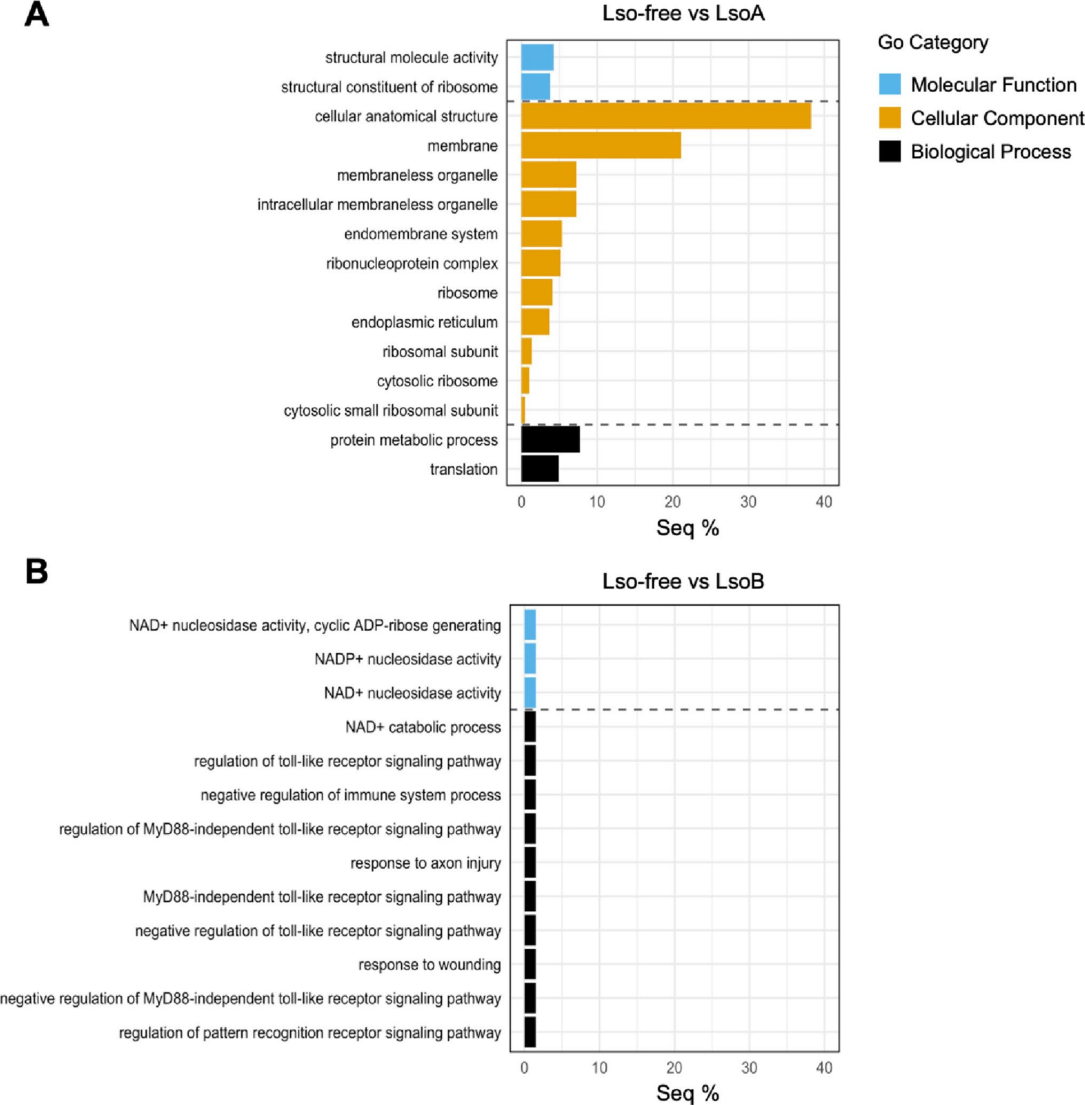
GO enrichment analysis of DEGs (**A**) GO terms enriched among the DEGs in the Lso-free vs. LsoA comparison after a 5-day AAP. (**B**) GO terms enriched among the DEGs in the Lso-free vs. LsoB comparison after a 5-day AAP. Bars represent the percentage of sequences assigned to each enriched GO term (Seq %) among the DEGs. GO enrichment was performed using the Blast2GO tool with default parameters, and significantly enriched terms were identified at FDR < 0.05. All the enriched GO terms by sequence percentage are shown

GO enrichment analysis was conducted using Fisher’s exact test (FDR < 0.05) to investigate the biological roles of the DEGs. Among the six comparisons, only Lso-free vs. LsoA and Lso-free vs. LsoB after a 5-day AAP had enriched GO terms. In the Lso-free vs. LsoA, 15 enriched GO terms were identified across cellular component, biological process, and molecular function, whereas in the Lso-free vs. LsoB, 13 enriched GO terms were found in biological process and molecular function. GO enrichment revealed distinct gut responses to LsoA and LsoB after a 5-day AAP. Specifically, in response to LsoA, enriched cellular component terms included “cell anatomical structure”, “membrane”, “intracellular membraneless organelle”, and “endoplasmic reticulum”. In addition, GO terms related to protein synthesis and translation, such as “ribosome”, “ribonucleoprotein complex”, “ribosomal subunit”, “cytosolic small ribosomal subunit”, and “structural constituent of ribosome” were enriched (Fig. 5A). In contrast, LsoB responses were characterized enrichment of GO terms associated with stress and immunity such as “response to wounding”, “negative regulation of immune system process”, “Myd88-independent toll-like receptor signaling pathway”, and “response to axon injury”, as well as “NAD + nucleosidase activity” and “NADP + nucleosidase activity” in the molecular function category (Fig. 5B).

### Validation of differentially expressed genes by Real-time quantitative PCR (RT-qPCR)

**Fig. 6:**
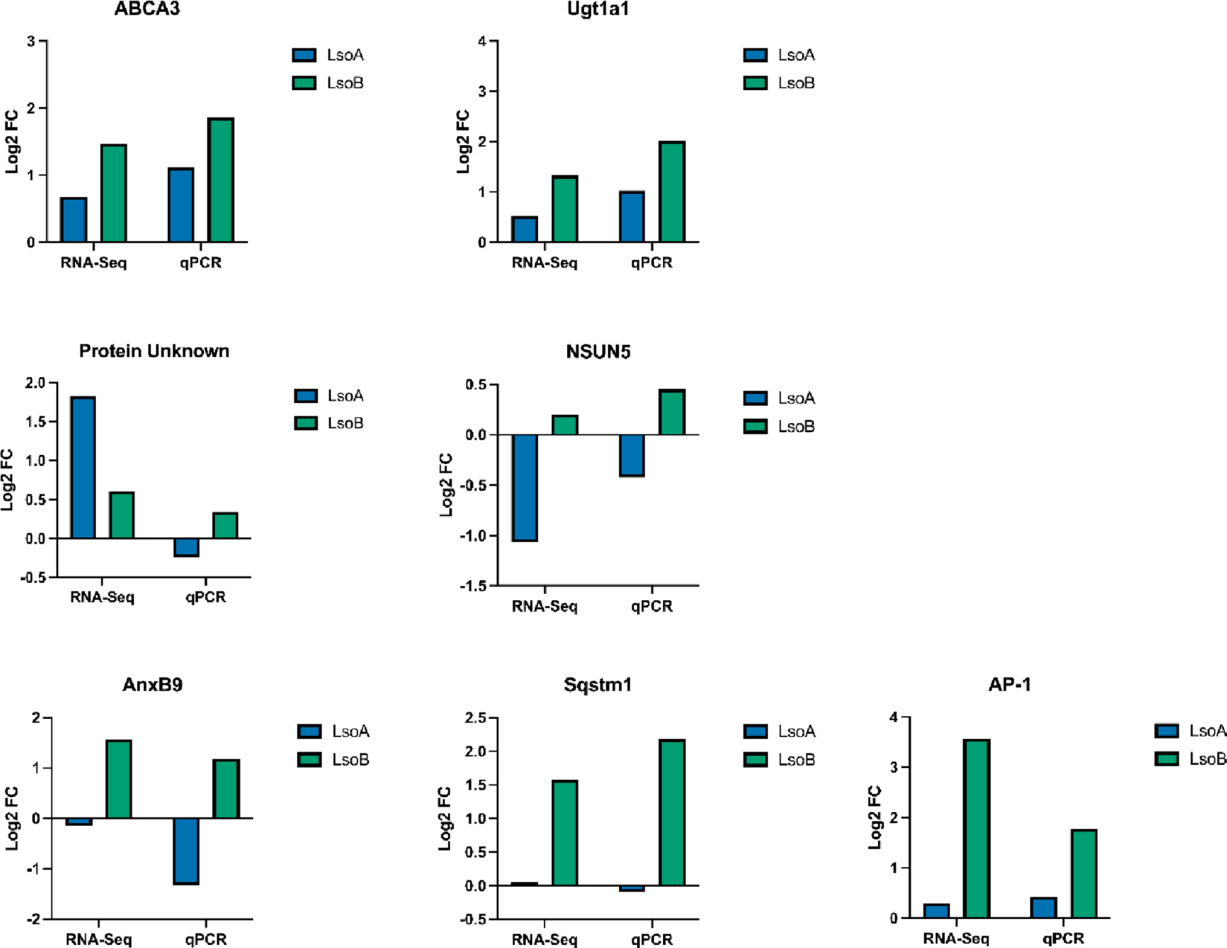
Comparison of gene expression profiles derived from the RNA-Seq and RT-qPCR analyses. The log_2_-transformed fold change (FC) gene expression from the transcriptomic data analysis (left side graphs) was calculated by Sleuth using the Lso-free treatment as a reference. For the RT-qPCR analyses (right side graphs), the log_2_-transformed FC was calculated using 2^^^-ΔΔCT method using the Lso-free treatment as reference

To validate the bioinformatic analyses, we selected seven genes identified as DEGs after a 5-day AAP and tested their expression using RNA obtained from independent samples compared to the RNA submitted for sequencing. The DEGs were selected from the intersection regions of the Venn diagram (Fig. 4). *ATP-binding cassette sub-family A member 3* (*ABCA3*) and *UDP-glucuronosyltransferase 1–1* (*Ugt1a1*) were selected from the bright yellow region which includes DEGs shared between the Lso-free vs. LsoA and Lso-free vs. LsoB comparisons; *protein of unknown function* and *28 S rRNA (cytosine-C(5))-methyltransferase* (*NSUN5*) were selected from the dark blue region which includes DEGs shared between the Lso-free vs. LsoA and LsoA vs. LsoB comparisons; *Annexin B9 (AnxB9)*, *Sequestosome-1* (*Sqstm1*), and *AP-1 (Jun)* were selected from the dark cyan region which includes DEGs shared between the comparisons between Lso-free vs. LsoB and LsoA vs. LsoB. Except for the *protein of unknown function*, the RT-qPCR results were consistent with the bioinformatic analyses (Fig. 6).

## Discussion

Studying pathogen accumulation dynamics in insect vectors helps understand disease epidemiology and identify transmission control strategies. Here, we measured the accumulation of LsoA and LsoB in nymphs following four AAPs and found that LsoA accumulation was lower than LsoB at each AAP. Similar results were obtained in adult guts upon acquisition [33]. However, these results differ from our previous analysis of the accumulation of LsoA and LsoB in the gut of nymphs from infected colonies in which we found that LsoA titer had plateaued by the third-instar and was higher than LsoB, but by the fifth-instar, LsoB titer had increased to similar levels to LsoA [19]. Our present results support hypothesis that lower LsoB titer in nymphs from infected colonies could be linked to a higher mortality of nymphs that acquired LsoB early in their development [16], the existence of some type of trans-generational immune priming [22], or that psyllid responses can differ between younger and older nymphs [34]. Indeed, differences in immunity between psyllid adults and nymphs have been reported by transcriptomic analysis [35] and proteomic profiling [36]. While most prior studies used whole body samples, our gut-specific transcriptome analysis adds insight into localized responses, which is important because the gut is a critical barrier for pathogen accumulation and transmission.

Consistent with the difference in acquisition, we also observed different transmission efficiency by nymphs following an 8-day AAP. LsoB was transmitted to plants as early as 17 days after the beginning of the acquisition, whereas LsoA transmission was first detected after 21 days. Because LsoB titer plateaued after a 5-day AAP while the LsoA titer remained low, we expected higher transmission efficiency of LsoB and we found 40% of LsoB-infected plants by day 17. These results indicate that LsoB had a shorter latent period, allowing earlier transmission and that this latency was shorter when the acquisition occurred during the nymphal stage than in adults [33]. No substantial increase in transmission efficiency was observed through day 29, suggesting that a transmission-capable threshold is reached before day 17, and this may also reflect the ability of LsoB to persist in the vector. In contrast, we observed a delay in LsoA transmission which might reflect slower accumulation. Because we used single nymphs for the transmission assay, overall transmission rates were relatively low, and unmonitored daily mortality may have led to underestimation of actual transmission rate (Table S3 and S4). Although our experiments were not designed to quantify mortality, higher mortality was observed when nymphs acquired LsoB than LsoA. From an epidemiological perspective, our results imply that older nymphs (fifth-instar) or adults could be the main life stages that transmit LsoB, while LsoA could be efficiently transmitted by nymphs or adults that acquired the pathogen during their nymphal development.

Lso is a phloem-limited pathogen and can only be acquired once the stylets reach the phloem. In adults, 2.5-hour-starved psyllids reach the phloem 4- to 5.5-h after the first probe [37]. Although this has not been measured in nymphs, Lso titer after a 1- and 5-day AAP suggest distinct stages of invasion: a relatively early stage in the invasion, characterized by increasing titers at 1-day AAP, and an advanced invasion after 5 days for LsoB but not yet for LsoA. Thus, we analyzed the gut transcriptome of nymphs after a 1- and 5-day AAP to capture early and later phases of colonization and to compare responses to each haplotype. We identified fewer DEGs following a 1-day AAP than a 5-day AAP, consistent with early and advanced infection. Our analysis also identified different transcriptional responses following feeding on LsoA- or LsoB-infected plants. There were no DEGs shared between Lso-free vs. LsoA and Lso-free vs. LsoB comparisons after a 1-day AAP and only 22 shared after a 5-day AAP. Interestingly, more DEGs were identified in the LsoB treatment after a 1-day AAP, whereas more genes were regulated in response to LsoA after a 5-day AAP. In fact, in this latter AAP, more DEGs were found in the LsoA vs. LsoB comparison than the Lso-free vs. LsoB, highlighting the distinct response elicited by the two haplotypes.

After a short AAP on LsoA-infected plants, most regulated genes (63.5%) were annotated as “Protein of unknown function” (Table S7). Among characterized genes, three up-regulated DEGs were annotated as protein-L-isoaspartate (D-aspartate) O-methyltransferase, repair enzymes that recognize and methylate damaged proteins containing L-isoaspartyl or D-aspartyl residues and are involved in the defense against bacteria [38]. Therefore, these genes could play an important role in the response to the early LsoA infection and could signal the initial stages of stress by protein damage. In the comparison between Lso-free and LsoB, there were four down-regulated genes encoding cuticular proteins (Table S8), suggesting a shift of resources from development to survival. Indeed, several immune-related genes were also regulated in the LsoB treatment. For example, down syndrome cell adhesion molecule-like protein, involved in the microbe recognition, was the most up-regulated gene [39]. Cystathionine beta-synthase was also up-regulated; this gene is involved in the expression of glutathione, which helps in reducing the oxidative stress that might be caused by LsoB infection [40].

After a 5-day AAP, genes involved in protein translation and endoplasmic reticulum associated degradation (ERAD) pathway, two major stress responses, were regulated in the Lso-free vs. LsoA comparison. There were 40 ribosomal genes down-regulated following AAP on LsoA-infected plants (Table S10), suggesting that LsoA could affect protein translation in the nymphal gut. Indeed, disruption of host protein synthesis is a prevalent tactic of various viral and bacterial pathogens [41, 42]. Several ERAD-related DEGs, including endoplasmic reticulum lectin 1, LON peptidase N-terminal domain and RING finger protein, Ubiquitin conjugating enzymes, ER degradation-enhancing alpha-mannosidase-like protein 2 (EDEM-2), and Selenoprotein F, were also regulated (Table S10). Furthermore, the calcium-transporting ATPase sarcoplasmic/endoplasmic reticulum type (SERCA), which regulates calcium homeostasis in the ER lumen, was expressed at higher levels in the gut of nymphs having fed on LsoA-infected plants (Table S10). Together, the regulation of ribosomal proteins and ER-stress response genes suggests a role of the ER in LsoA infection. ERAD was also reported in the carrot psyllid nymphs reared on celery plants infected with LsoD, where genes involved in ERAD pathway were regulated [43]. The identification of similar regulations in response to LsoA and LsoD is not surprising considering that LsoA is more similar to LsoD than to LsoB at the nucleotide identity level [44].

We also observed the down-regulation of the genes involved in cell cycle and differentiation, such as centriolar coiled-coil protein of 110 kDa, muscle LIM protein Mlp84B%2 C isoform X3, and serine/threonine-protein kinase PRP4 homolog, and genes involved in chromatin remodeling, such as deoxynucleotidyl transferase terminal-interacting protein 2 and transcriptional regulator ATRX-like in the Lso-free vs. LsoA comparison after a 5-day AAP (Table S10). These results suggest that LsoA invasion affects cell cycle and differentiation in the nymphal gut.

In the LsoB treatment after a 5-day AAP, we found the up-regulation of the genes linked to apoptosis, such as cell division cycle and apoptosis regulator protein 1, as well as sequestosome-1, a selective autophagy receptor that is typically degraded as the autophagic flux progresses [45] (Table S11). Conversely, in adults exposed to LsoB, autophagy-related genes were up-regulated while apoptosis-related genes were down-regulated [46], suggesting stage-specific responses. However, our recent study found no evidence of autophagy activation in the guts of LsoB-infected adults [47], indicating that transcriptional changes do not always correspond to pathway activation. Whether these responses are functionally induced in nymphs remains to be tested.

We also found up-regulated DEGs involved in humoral responses, such as cytokine signaling 2 and transcription factor AP-1, which regulate the JAK/STAT and JNK pathways, respectively (Table S11). The JAK/STAT pathway has been studied in virus-vector interactions [48, 49], so we cannot exclude the possibility that humoral responses contribute to psyllid defense against LsoB, particularly through the JAK/STAT and JNK pathways. Although these pathways are mainly known to be involved in immunity, they also play essential roles in epithelial renewal by removing damaged cells and stimulating stem cell proliferation and differentiation [50]. Thus, their regulation in response to LsoB may reflect both immune response and gut renewal, especially if apoptosis or autophagy are induced. Such responses, together with possible tissue damage caused by LsoB or a reallocation of resources from development to immunity, may help explain the increased in nymphal mortality associated with LsoB infection.

## Conclusion

Similar to potato psyllid adults, LsoB accumulated faster in nymphs and was transmitted more efficiently than LsoA. These results suggest the induction of different molecular responses to each haplotype. Specifically, LsoA and LsoB significantly affected the physiology of the nymphal gut resulting in changes in the expression of genes related to stress response, immunity, epithelial renewal, and cell repair/cycle after a 5-day AAP with distinct transcriptional regulation induced by LsoA and LsoB. These psyllid responses are probably linked to the different infection strategies used by these two haplotypes: LsoB appears to be more aggressive inducing immune responses and causing higher psyllid mortality, while LsoA accumulates slowly inducing fewer immune responses. Our study sheds light on the different accumulation and transmission patterns and molecular interactions between the two Lso haplotypes and potato psyllid nymphs; this information can help understand different epidemiological aspects of LsoA and LsoB vectored by psyllid nymphs.

## Supplementary Information

Below is the link to the electronic supplementary material.

Supplementary Material 1

Supplementary Material 2

Supplementary Material 3

## Data Availability

The datasets generated and/or analyzed during this study are available in the NCBI-SRA database under BioProject: PRJNA1332807 (temporary submission ID is: SUB15656931). The BioSample accessions are: SAMN51761342, SAMN51761343, SAMN51761344, SAMN51761345, SAMN51761346, SAMN51761347, SAMN51761348, SAMN51761349, SAMN51761350, SAMN51761351, SAMN51761352, SAMN51761353, SAMN51761354, SAMN51761355, SAMN51761356, SAMN51761357, SAMN51761358, SAMN51761359.
