# Supplementary material for: Accumulation and transmission dynamics of ‘Candidatus liberibacter solanacearum’ haplotypes A and B by potato psyllid nymphs: bioassay and transcriptomic insights
Source: Mol Biol Rep. 2026 Jan 6;53(1):261. doi: 10.1007/s11033-025-11417-y (PMC12775102; doi:10.1007/s11033-025-11417-y)
Supplement: Supplementary file 2 — Supplementary Material 2 [file 11033_2025_11417_MOESM2_ESM.docx]

Table S2. Primers for RT-qPCR validation

| Genes | Primers |
| --- | --- |
| ABCA3 | F: AAGTTTGCCGTGGGCTATCA |
|  | R: TGGAAGGTGACCATGCCTTC |
| Unknown protein | F: CTCGTCTCCTACTTCGACGC |
|  | R: GAGGTGTCTTCTTGGTCGCA |
| NSUN5 | F: AGAACGAGCAAGTCGTGGAG |
|  | R: TCCTACCATGCGCGTACTTC |
| AnxB9 | F: GGCAGGACACGACATAGAGG |
|  | R: TGCCATAGCGTCCTTCAGTC |
| Sqstm1 | F: CACAAGAGACACAAGGGGCT |
|  | R: CGAGGCTACGCTACCAGATG |
| AP-1 | F: GATGGCTGCTCCTCCATCTAC |
|  | R: TTCGAGCTTGATGCGCTCTT |
| Ugt1a1 | F: CCCTCCACACAATCCATGCT |
|  | R: TTCCAATATGCAACGGGCCT |

Table S3. LsoA and LsoB transmission by a potato psyllid nymph (experiment 1).

|  | **Day Post-Transfer** | | | | |
| --- | --- | --- | --- | --- | --- |
| **LsoA (experiment 1)** | **Plant** | **17** | **21** | **25** | **29** |
|  | **1** | Alive | Alive | Alive | Alive |
|  | **2** | Alive | Alive | Alive | Alive |
|  | **3** | Alive | Alive | **D/R** | Alive |
|  | **4** | Alive | Alive | Alive | Alive |
|  | **5** | Alive | Alive | Alive | Alive |
|  | **6** | Alive | **D/R** | Alive | Alive |
|  | **7** | Alive | Alive | Alive | Alive |
|  | **8** | Alive | Alive | Alive | Alive |
|  | **9** | Alive | Alive | Alive | Alive |
|  | **10** | Alive | Alive | Alive | Alive |
| **LsoB (experiment 1)** | **Plant** | **17** | **21** | **25** | **29** |
|  | **1** | Alive | **D/R** | **D/R** | Alive |
|  | **2** | Alive | Alive | Alive | Alive |
|  | **3** | Alive | **D/R** | Alive | Alive |
|  | **4** | Alive | Alive | **D/R** | Alive |
|  | **5** | Alive | Alive | Alive | Alive |
|  | **6** | Alive | **D/R** | Alive | Alive |
|  | **7** | Alive | **D/R** | Alive | Alive |
|  | **8** | **D/R** | Alive | Alive | Alive |
|  | **9** | Alive | Alive | **D/R** | Alive |
|  | **10** | Alive | Alive | Alive | Alive |

A third-instar nymph following a 8-day of AAP was given sequential inoculation. There was a total of 4 sets of plants with 10 plants each. Red indicates Lso-infected plants, Alive indicates the psyllid was alive at the corresponding timepoint and D/R (dead/replaced) indicates that the psyllid was dead and replaced.

Table S4. LsoA and LsoB transmission by a potato psyllid nymph (experiment 2).

|  | **Day Post-Transfer** | | | | |
| --- | --- | --- | --- | --- | --- |
| **LsoA (experiment 2)** | **Plant** | **17** | **21** | **25** | **29** |
|  | **1** | Alive | **D/R** | Alive | Alive |
|  | **2** | **D/R** | Alive | **D/R** | Alive |
|  | **3** | Alive | Alive | Alive | **D** |
|  | **4** | **D/R** | Alive | Alive | **D** |
|  | **5** | Alive | Alive | Alive | Alive |
|  | **6** | Alive | Alive | Alive | Alive |
|  | **7** | Alive | Alive | Alive | **D** |
|  | **8** | Alive | Alive | Alive | Alive |
|  | **9** | Alive | Alive | Alive | **D** |
|  | **10** | Alive | Alive | Alive | Alive |
| **LsoB (experiment 2)** | **Plant** | **17** | **21** | **25** | **29** |
|  | **1** | Alive | Alive | Alive | Alive |
|  | **2** | Alive | Alive | **D/R** | Alive |
|  | **3** | Alive | Alive | Alive | **D** |
|  | **4** | Alive | **D/R** | Alive | **D** |
|  | **5** | Alive | Alive | Alive | Alive |
|  | **6** | **D/R** | Alive | **D/R** | **D** |
|  | **7** | Alive | Alive | Alive | Alive |
|  | **8** | Alive | Alive | Alive | Alive |
|  | **9** | **D/R** | Alive | Alive | Alive |
|  | **10** | Alive | Alive | Alive | Alive |

A third-instar nymph following a 8-day of AAP was given sequential inoculation. There was a total of 4 sets of plants with 10 plants each. Red indicates Lso-infected plants, Alive indicates the psyllid was alive at the corresponding timepoint, D/R (dead/replaced) indicates that the psyllid was dead and replaced, D (dead) indicates that the psyllid was dead and was not replaced because it was the last time point.

Table S5. Odds ratio and Fisher’s exact test *P*-value

| LsoA vs. LsoB | Odds Ratio | 95% CI Lower | 95% CI Upper | *P*-value |
| --- | --- | --- | --- | --- |
| Day 17 | 0.036 | 0.002 | 0.677 | 0.003 |
| Day 21 | 0.381 | 0.074 | 1.964 | 0.243 |
| Day 25 | 0.122 | 0.006 | 2.527 | 0.109 |
| Day 29 | 1.000 | 0.283 | 3.534 | 1 |

Table S6. Summary statistics of transcriptome libraries.

| Sample | Reads_mapped | Reads_processed | Mapped (%) |
| --- | --- | --- | --- |
| Lso-free 1-day rep1 | 12639436 | 22300608 | 0.5668 |
| Lso-free 1-day rep2 | 12155928 | 21965078 | 0.5534 |
| Lso-free 1-day rep3 | 12501348 | 22805294 | 0.5482 |
| Lso-free 5-day rep1 | 12191541 | 22491098 | 0.5421 |
| Lso-free 5-day rep2 | 10960818 | 22473326 | 0.4877 |
| Lso-free 5-day rep3 | 12542304 | 25617707 | 0.4896 |
| LsoA 1-day rep1 | 15052461 | 23335858 | 0.645 |
| LsoA 1-day rep2 | 13344610 | 23302791 | 0.5727 |
| LsoA 1-day rep3 | 12624459 | 23310590 | 0.5416 |
| LsoA 5-day rep1 | 14039326 | 23289197 | 0.6028 |
| LsoA 5-day rep2 | 12844607 | 22904353 | 0.5608 |
| LsoA 5-day rep3 | 14622707 | 25757720 | 0.5677 |
| LsoB 1-day rep1 | 11250331 | 20604740 | 0.546 |
| LsoB 1-day rep2 | 11810608 | 22335048 | 0.5288 |
| LsoB 1-day rep3 | 13998291 | 24798736 | 0.5645 |
| LsoB 5-day rep1 | 13108676 | 22441144 | 0.5841 |
| LsoB 5-day rep2 | 13473849 | 27761542 | 0.4853 |
| LsoB 5-day rep3 | 11673824 | 22325272 | 0.5229 |
